# L-Citrulline in Neonates: From Bench to Bed Side

**DOI:** 10.3390/children12010042

**Published:** 2024-12-30

**Authors:** Dwayne Mascarenhas, Atefeh Mohammadi, Randa Higazy, Julijana Ivanovska, Estelle Gauda, Bonny Jasani

**Affiliations:** 1 Division of Neonatology, The Hospital for Sick Children, Toronto, ON M5G 1X8, Canada; dwayne.mascarenhas@sickkids.ca (D.M.); estelle.gauda@sickkids.ca (E.G.); 2 Translational Medicine and Cell Biology Programs, Peter Gilgan Centre for Research and Learning, The Hospital for Sick Children, Toronto, ON M5G 1E8, Canada; atefeh.mohammadi@sickkids.ca (A.M.); julijana.ivanovska@sickkids.ca (J.I.); 3 Department of Laboratory Medicine and Pathobiology, University of Toronto, Toronto, ON M5S 3K3, Canada; randa.higazy@sickkids.ca

**Keywords:** L-arginine, L-citrulline, nitric oxide, bronchopulmonary dysplasia, pulmonary hypertension, necrotizing enterocolitis

## Abstract

L-citrulline (L-CIT), a precursor to L-arginine (L-ARG), is a key contributor to the nitric oxide (NO) signaling pathway. Endothelial dysfunction, characterized by deficient nitric oxide synthesis, is implicated in the pathogenesis of various neonatal conditions such as necrotizing enterocolitis (NEC) and bronchopulmonary dysplasia (BPD) associated pulmonary hypertension (PH). This review summarizes the current evidence around the possible role of L-CIT supplementation in the treatment of these conditions. Detoxification of endogenously produced superoxide radicals is inadequate in preterm infants due to immature antioxidants that leads to the production of peroxynitrite, a reactive oxygen-free radical that is cytotoxic and causes damage to organelles and cellular membranes, further disrupting the coupling of endothelial NO synthase enzyme and the generation of high levels of reactive nitrogen and oxygen species. Animal studies in lipopolysaccharide-induced models of chorioamnionitis and hyperoxia- and inflammation-induced BPD-PH in rodent lung models revealed that L-CIT supplementation significantly mitigated structural changes in the pulmonary vasculature, preserved alveolar growth, and increased vascular endothelial growth factor gene expression, highlighting the anti-inflammatory and antioxidant effects of L-CIT supplementation. Similar benefits were noted in newborn piglet models of chronic hypoxia-induced PH and NEC. Pharmacokinetic studies in neonates have shown doses of 100–300 mg/kg/day to be safe and well tolerated. A few studies have shown the beneficial effects of L-CIT supplementation in pulmonary hypertension secondary to congenital heart disease, but evidence of efficacy in the neonatal population is lacking. While L-CIT shows promise in the treatment of various neonatal conditions, adequately powered studies to evaluate the safety and efficacy of L-CIT supplementation post-surgical NEC and BPD ± PH in the extremely preterm population are needed to translate this novel therapy to clinical practice.

## 1. Introduction

L-citrulline (L-CIT) is a non-essential amino acid and an important intermediate in the urea and nitric oxide (NO) cycles. NO plays an important role in the pulmonary vasculature and smooth muscle relaxation mediated by cyclic guanosine monophosphate (cGMP) leading to vasodilation [1]. Deficiency in endogenous substrates such as L-CIT and L-arginine (L-ARG) can lead to reduced NO production, which may play an important role in the pathophysiology of many diseases including pulmonary hypertension (PH) associated with bronchopulmonary dysplasia (BPD) and congenital heart disease (CHD). Not only does NO promote angiogenesis and alveolarization, but it is also important for normal gastrointestinal (GI) function. Insufficient NO in the gut leads to vasoconstriction of intestinal vessels, causing ischemia and increasing predisposition to necrotizing enterocolitis (NEC), a major neonatal gastrointestinal disease with a high burden of morbidities and mortality [2,3].

Therapeutic approaches that enhance NO signaling in the pulmonary vasculature and endothelium have long been considered and investigated as ideal targets in treating various neonatal diseases, primarily BPD-PH. This includes different sources of exogenous NO or NO donors and inhaled NO (iNO), however they are associated with significant side effects. NO donors, such as sodium nitroprusside (SNP) and sildenafil, cause significant systemic hypotension, accumulation of toxic metabolites (free radicals), and are limited by non-specific vasodilation [4]. L-Arg, the precursor to NO, has also been considered a therapeutic candidate; however, it undergoes a significant first-pass effect and is not well-tolerated, leading to a limited increase in NO levels and GI side effects in infants [5,6]. Many of these candidate pharmacotherapeutic agents have not been tested adequately or sufficiently, remaining unapproved and often used off-label. iNO is the only FDA-approved therapy for persistent PH of the newborn. However, approximately 40% of infants develop resistance and do not respond to iNO [7]. This highlights that there is an urgent need for an efficacious and safe therapy for treatment of chronic PH especially in extremely preterm neonates.

L-CIT, the precursor to L-Arg, emerges as a promising candidate to effectively and safely enhance NO signaling. This review summarizes the metabolism and pharmacokinetics of L-CIT, as well as its role in NO signaling, including all preclinical and clinical studies investigating L-CIT supplementation in neonates, highlighting its potential therapeutic benefit in various common neonatal conditions.

## 2. L-Citrulline: An Intermediate in the Urea and Nitric Oxide Cycles

**Figure 1:**
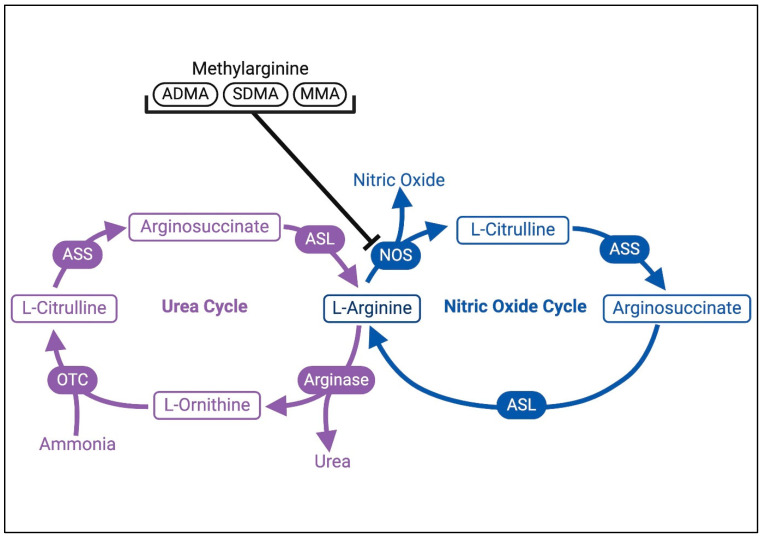
L-citrulline is an intermediate in the urea and nitric oxide cycles. L-arginine (L-ARG) is converted into L-citrulline (L-CIT) and nitric oxide (NO) by NO synthase (NOS). There are 3 forms of methylarginine that are endogenous inhibitors of NOS: Asymmetric Dimethylarginine (ADMA), Symmetric Dimethylarginine (SDMA), and Monomethylarginine (MMA). L-CIT is recycled back into L-ARG by Arginosuccinate Synthase (ASS) and Arginosuccinate Lyase (ASL). As part of the urea cycle, Arginase competes with NOS for the substrate, L-ARG, which can be converted into L-ornithine and urea. L-ornithine can be broken down into L-CIT by Ornithine Transcarbamylase (OTC).

L-CIT is produced endogenously by the small intestine from glutamine, glutamic acid, and proline. In the urea cycle, ornithine carbamoyl-transferase converts ornithine, a metabolite of the amino acid glutamine, into L-CIT (Figure 1). In the NO cycle, L-Arg is converted into L-CIT and NO by NO synthase (NOS) (Figure 1). L-CIT can then be recycled back into L-Arg by the enzymes arginosuccinate synthase (ASS) and arginosuccinate lyase (ASL), thereby increasing the bioavailability of the substrate for endogenous NO production [8].

Exogenous sources of L-CIT are limited, as the normal adult diet contains almost no L-CIT, except for watermelon, which has approximately 1.67–3.1 mg/g of fresh weight [9]. Although L-ARG is found in pediatric total parenteral nutrition or infant formulas, L-CIT is not a constituent, and its content in human milk is negligible [10]. In contrast to L-Arg, enterally absorbed L-CIT passes through the liver without major first-pass metabolism. L-CIT is not broken down by hepatic or intestinal arginase enzymes, resulting in higher bioavailability and longer circulation time [11]. Various neutral amino acid transporters play a role in transporting circulating L-CIT into vascular endothelial cells, which is subsequently converted into L-ARG by an enzymatic process [10]. The kidney, the main organ of L-CIT consumption, converts approximately 75% of circulating L-CIT into L-ARG in the proximal tubules and then releases it into circulation [1,10]. Urinary excretion of L-CIT accounted for less than 1% of the enteral L-CIT load filtered in the kidney [12]. In addition to renal conversion, L-CIT is also converted to L-ARG as part of the urea cycle, as shown in Figure 1. Infants born prematurely have a limited ability to produce L-ARG endogenously and rely on exogenous dietary sources of L-ARG or its precursors to maintain adequate circulating levels [13].

## 3. The Role of NOS and NO Signaling in Neonatal Diseases

Endothelial dysfunction is an important factor in the pathogenesis of acute and chronic diseases of prematurity, including intraventricular hemorrhage, retinopathy of prematurity, hypoxic-ischemic encephalopathy, PH, BPD, acute kidney injury (AKI), and NEC—essentially all the morbidities that occur in premature infants [14,15].

Endothelial cells line all blood vessels and play a critical role in health and disease by regulating the blood flow to organs and tissues. These cells are highly active and interact with soluble mediators that modulate inflammation, vascular permeability, coagulation balance, angiogenesis, and vascular tone [16,17]. When exposed to inflammation, oxidative and shear stress, the vascular endothelium shifts towards a proinflammatory, procoagulant, and vasoconstrictive phenotype, resulting in endothelial dysfunction [18].

The regulation of blood flow requires blood vessels to dilate and constrict to match oxygen and nutrition delivery with demand. The main regulator of vessel tone is NO that is released from cells, specifically vascular endothelial cells. NO is a gaseous, highly diffusible signaling molecule that binds to effectors on vascular smooth muscle cells to cause vasodilation [19,20]. NOS, the enzyme responsible for NO generation, exists in three isoforms: endothelial (eNOS), neuronal (nNOS), and inducible (iNOS). nNOS and eNOS are calcium-dependent and produce low levels of NO whereas iNOS, present in neutrophils and natural killer cells, is calcium-independent and produces large amounts of NO [20,21].

**Figure 2:**
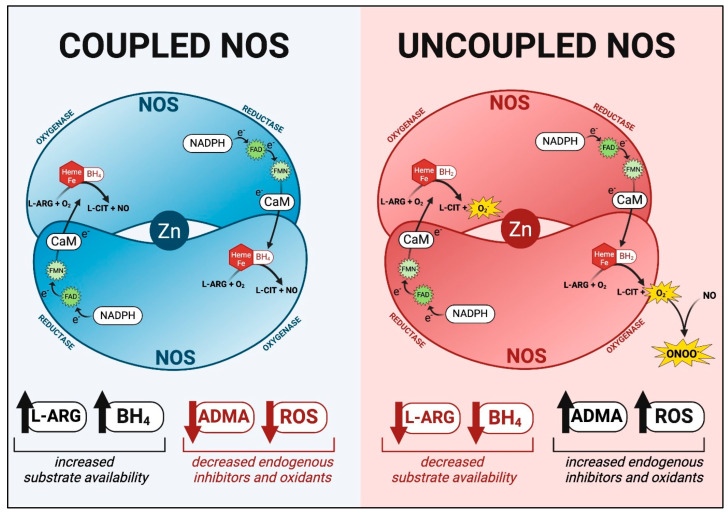
NOS coupling and uncoupling. Each NOS enzyme consists of an oxygenase and reductase domain that bind together to produce a coupled NOS homodimer. NOS can remain coupled in the presence of increased L-ARG and tetrahydrobiopterin (BH_4_) substrate availability and decreased presence of endogenous NOS inhibitors (ADMA, SDMA, and MMA), and decreased reactive oxygen species (ROS) production. The NOS homodimer becomes uncoupled in unfavorable conditions such as decreased L-ARG and BH_4_ substrate availability, an increased presence of endogenous NOS inhibitors, and increased ROS production. ROS, such as superoxide anions, can react with NO to produce peroxynitrite, a potent-free radical that can cause widespread damage.

NOS is an oxi-reductase enzyme consisting of homodimer proteins that bind together. The enzymatic process uses molecular oxygen, L-ARG, heme, zinc, and tetrahydrobiopterin (BH_4_), an essential cofactor for the enzyme, to produce L-CIT and NO. This process is called eNOS coupling (Figure 2). In addition to making L-CIT and NO, NADPH is reduced, and molecular oxygen is oxidized generating superoxide. Superoxide anions can be detoxified by the antioxidant superoxide dismutase (SOD) to make H_2_O_2_ and oxygen. The detoxification of superoxide radicals is very important, as NO can react with superoxide to produce peroxynitrite, a reactive oxygen radical that is cytotoxic and causes damage to organelles and cellular membranes [22,23,24,25]. Premature infants have low levels of SOD and other antioxidants, and high levels of oxidants that disrupt the coupling of eNOS. When eNOS is uncoupled, high levels of reactive nitrogen (RNS) and reactive oxygen species (ROS) are generated (Figure 2).

Cellular factors that lead to uncoupled eNOS include high levels of ROS, endogenous NOS inhibitors such as asymmetric dimethylarginine (ADMA), or elevated enzymes that compete for the same substrate, L-ARG, such as arginase I and II (Figure 2). In addition, low levels of the essential cofactor BH_4_ and the substrate L-ARG, all lead to eNOS uncoupling and decreased NO bioavailability (Figure 2). These factors are elevated during inflammation, oxidative stress, intermittent hypoxia, prenatal or postnatal undernutrition and overnutrition, and AKI that commonly occur in premature infants. The resulting decrease in NO bioavailability exacerbates endothelial dysfunction, causing acute and chronic organ damage with associated co-morbidities [15].

L-CIT can restore eNOS coupling in pulmonary artery endothelial cells induced by hypoxia by increasing the bioavailability of its substrate, L-ARG [24]. In newborn preclinical models of cardiovascular and chronic kidney diseases, concurrent supplementation with L-CIT during late gestation and early postnatal development mitigates biomarkers of oxidative stress and maintains L-ARG and NO bioavailability [26]. In our preclinical rodent model of postnatal growth restriction, our preliminary data consistently show that concurrent treatment with L-CIT during the first 3 weeks of postnatal development with nutritional deprivation mitigates upregulation of pro-inflammatory cytokine gene and protein expression in the lung, pancreas, and blood, and prevents histopathological changes in the lung and heart consistent with PH [27,28]. L-CIT, as a source of L-ARG, improves endothelial dysfunction in adults, children, and newborn animal models discussed in the sections below on preclinical and clinical studies. Thus, L-CIT supplementation may have a therapeutic role in preterm infants where the underlying pathophysiology involves uncoupled NOS secondary to ongoing inflammation and oxidative stress.

## 4. Pharmacokinetics of L-Citrulline

Currently, there is limited data on the pharmacokinetics (PK) of L-CIT in the pediatric and neonatal population. Barr et al. evaluated the PK of a single dose of intravenous (IV) L-CIT in 17 children under the age of six years who were undergoing cardiopulmonary bypass for cardiac surgery [29]. In the first phase, eight patients received two doses of IV L-CIT, one dose immediately after initiation of cardiopulmonary bypass and another dose four hours later at the time of admission to the intensive care unit, in a dose escalation manner. The first two patients received 50 mg/kg and had a peak L-CIT level of 220 μmol/L and a 4 h trough level of 40 μmol/L. The next two patients received 100 mg/kg and had peak and 4 h trough levels of 375 μmol/L and 50 μmol/L, respectively. The last four patients received 150 mg/kg of IV L-CIT and had peak L-CIT concentrations of 600 μmol/L and 4 h trough levels of 80 μmol/L. As the half-life calculated was found to be only 60 min, they proceeded to phase 2, which enrolled an additional nine patients. Based on PK simulations from phase 1 data, these children received a 150 mg/kg IV bolus followed by a continuous infusion of 9 mg/kg/h starting 4 h after the bolus. The post-operative mean plasma L-CIT concentrations achieved a steady state of 150–250 μmol/L during the 48 h study period, with an estimated L-CIT clearance of 0.52 ± 0.28 L/kg·h.

In neonates, Fike et al., who studied the PK of a single oral dose of L-CIT (150 mg/kg) given to ten extremely preterm infants at 32 ± 1 weeks post-menstrual age [30], reported their findings. Time windows were used to maximize sampling, plasma L-ARG and L-CIT concentrations were assessed by non-compartmental analysis, and a simulation-based methodology was used to derive optimal dosing strategies. The simulated doses of 37.5 mg/kg given every 6 h (150 mg/kg/day) were noted to produce a steady-state concentration of around 50 μmol/L, with a volume of distribution, clearance, and half-life of 302.89 mL, 774.96 mL/h, and 16 min, respectively. The area under the curve from the time of dosing to the time of last concentration was 1473.3 μmol·h/L, and the maximum observed concentration and time to maximum observed concentration were noted to be 799 μmol/L and 1.55 h, respectively.

The same research group subsequently studied the effect of multi-dose regimen of enteral L-CIT on six preterm infants born ≤ 28 weeks’ gestation [31]. Each infant received 60 mg/kg/dose at 6 hourly intervals (240 mg/kg/day) for 3 days via a nasogastric tube half an hour prior to feeding. For the majority of the infants, the trough L-CIT levels prior to the last dose were 10–81% higher than the baseline concentration prior to supplementation, with two infants measuring trough levels that exceeded 50 μmol/L.

## 5. Preclinical Studies in Neonatal Animals

The use of newborn animal models to increase our understanding of neonatal diseases and support the discovery of novel therapeutics to improve the standard and quality of care for neonates has been crucial. Preclinical studies in the literature have reported the effects of L-CIT supplementation in various animal models of adult, pediatric, and neonatal diseases.

**Figure 3:**
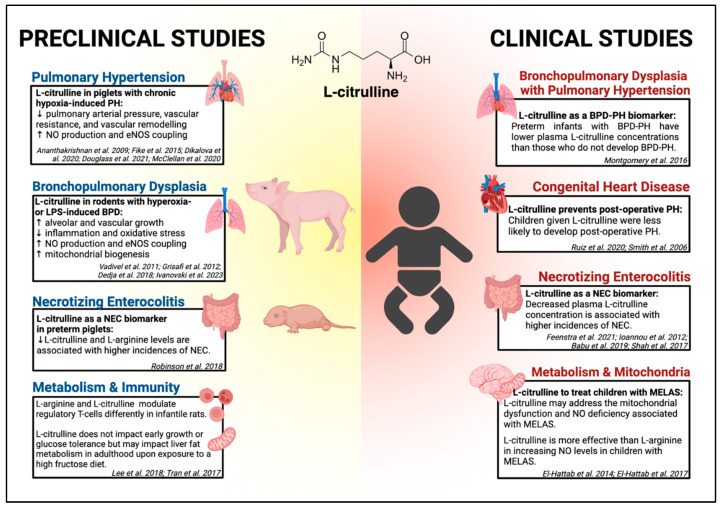
A summary of preclinical and clinical studies investigating L-citrulline supplementation in neonates.

Gaining insights into the developmental changes in the renal expression and activity of enzymes necessary for L-CIT metabolism in neonatal pigs, ASS and ASL, is key to understanding the potentially beneficial effects of L-CIT supplementation in neonates. In a recent study, Mohammad et al. reported that the expression and activity of these key enzymes are lower in neonates than in juvenile animals; however, the capacity to metabolize L-CIT remains unaffected [32]. Despite the reduced activity of ASS and ASL in neonates, there is still excess capacity for L-CIT metabolism. Infants of all ages, including preterm, are successfully able to metabolize endogenously produced and exogenously supplemented CIT, consequently increasing L-ARG bioavailability. In this section, we summarize the outcomes associated with L-CIT supplementation in various neonatal animal models of PH, BPD, NEC, and other uses (Figure 3).

### 5.1. Preclinical Studies: Neonatal Lung Disease

BPD is characterized by fewer and abnormal alveoli leading to less surface area for gas exchange and fewer and abnormal pulmonary blood vessels, which may lead to PH. BPD is a consequence of lung inflammation during the canalicular and saccular stages of lung development, induced by increased levels of pro-inflammatory cytokines and oxidative stress, initiating injury to cell membranes, mitochondria, and other organelles, causing cell death and maldevelopment [33,34,35,36,37].

L-CIT supplementation has been studied in hyperoxia and inflammation-induced models of neonatal lung injury and BPD. In hyperoxia-induced models of BPD-PH, newborn rodents exhibited decreased alveolarization and angiogenesis, as well as right ventricular hypertrophy [38,39]. L-CIT treatment during hyperoxia exposure significantly mitigated structural changes in the pulmonary vasculature, preserved alveolar growth, and increased vascular endothelial growth factor (VEGF) gene expression [38,39]. Plasma concentrations of L-ARG and L-CIT were significantly lower in the hyperoxia-exposed animals and improved with L-CIT treatment [38].

Dedja et al. explored the effect of L-CIT in newborn rats in a lipopolysaccharide (LPS)-induced model of chorioamnionitis and postnatal lung injury [40]. In the LPS injury group, newborn rat lungs displayed arrested alveolarization, which was improved with L-CIT supplementation. In our recent study, we investigated the effect of postnatal LPS-induced lung inflammation in newborn rats. We reported the extensive anti-inflammatory and antioxidant effects of L-CIT supplementation as well as its ability to preserve mitochondrial health [41]. These beneficial effects of L-CIT were linked to two signaling pathways, the peroxisome proliferator-activated receptor gamma coactivator-1 alpha (PGC-1α) and sirtuin 1 (SIRT1). These two principal regulators control antioxidant response by activating SODs, regulating nuclear respiratory factor 1 (NRF1) and mitochondrial transcription factor A (TFAM), which preserve the mitochondria, and inhibiting NFκB signaling to decrease the levelinflammatory cytokines and chemokines.

### 5.2. Preclinical Studies: Neonatal Pulmonary Hypertension

Fike et al. have conducted extensive studies investigating the effect of L-CIT supplementation on neonatal animals with PH, making significant contributions to this field [42]. Insufficient L-ARG availability and consequent NO production lead to vascular dysfunction and PH [43]. Studies to date have shown that L-CIT mitigates PH through improved NO signaling in neonatal animals.

Newborn piglets exposed to chronic hypoxia develop PH and have reduced NO production. Oral L-CIT supplementation, immediately prior to and continued throughout the duration of the hypoxic exposure, mitigates the development of PH and significantly increases pulmonary NO production [44]. Specifically, both pulmonary arterial pressure and pulmonary vascular resistance were lower in the chronically hypoxic animals treated with L-CIT compared to the untreated hypoxic animals. L-CIT supplementation ameliorates the chronic hypoxia-induced structural changes in pulmonary circulation, such as pulmonary vascular remodeling and lung capillary formation, mediated by VEGF signaling [45].

Fike et al. have also conducted investigations using L-CIT as a rescue treatment after the onset of injury since this better recapitulates the potential clinical use of L-CIT [46]. L-CIT decreased pulmonary vascular resistance in hypoxic piglets, improved NO signaling including increased NO production, reduced superoxide production, and improved eNOS coupling. This is important as eNOS uncoupling contributes to impaired NO signaling and, subsequently, disease pathophysiology in a newborn piglet model of chronic hypoxia-induced PH [47,48]. L-ARG supplementation has demonstrated some beneficial but inconsistent effects in encouraging eNOS recoupling [49,50,51]. However, L-CIT, the precursor to L-ARG, presents an alternate strategy in recoupling eNOS [52,53].

Recently, Fike and colleagues have studied methods to increase the efficacy of L-CIT supplementation through combination therapy, particularly using BH_4_ and folic acid [24,54]. These studies determined that co-treatment with L-CIT and BH_4_, or L-CIT and folic acid in chronically hypoxic newborn piglets reduced pulmonary vascular resistance and increased eNOS coupling more than in piglets with either treatment alone.

### 5.3. Preclinical Studies: Neonatal Gut Disease

In a preclinical study conducted by Robinson et al., L-CIT and L-ARG fluxes were measured in preterm piglets, showing that lower production rates were associated with higher incidences of NEC [55]. The results from this study suggest that enhancing L-CIT and L-ARG availability may be a preventive strategy for NEC in neonatal care.

### 5.4. Other Preclinical Studies

L-CIT, beyond its established roles in NO production, vascular health, lung disease, and gut health, exhibits diverse effects on immune function and metabolic regulation. Lee et al. investigated the programming effects of L-ARG and L-CIT supplementation on regulatory T-cell function in infantile rats. Both supplements were found to modulate regulatory T-cell (Treg) immune effects by increasing interleukin-10 (IL-10) levels, an anti-inflammatory cytokine [56]. However, only L-CIT supplementation resulted in an elevation of transforming growth factor-beta 1 (TGF-β1) levels and modulated FOXP3 expression, indicating distinct impacts on Treg function compared to L-ARG. These studies reviewed above highlight the multifaceted effects of L-CIT supplementation, underscoring its potential as a therapeutic agent in various physiological contexts and neonatal diseases.

## 6. Clinical Studies in Pediatric and Neonatal Populations

In this section, we summarize the outcomes associated with L-CIT supplementation in clinical studies of BPD-PH, CHD, NEC, and metabolic syndrome in pediatric or neonatal populations (Figure 3).

### 6.1. Safety and Tolerability

L-CIT has the potential to lower the systemic arterial blood pressure and has been associated with GI disturbances in adults when administered orally. However, infants and children with CHD who received intravenous L-CIT (150 mg/kg) in the perioperative period were noted to be hemodynamic stable with no observed hypotension [29]. A study in six preterm infants on full feeds who received 12 doses of 60 mg/kg enteral L-CIT at 6 hourly intervals (total 240 mg/kg/day) also showed good enteral tolerance to the drug with no episodes of hypotension requiring intervention [31]. A pilot study on enteral supplementation in 42 preterm infants ≤ 33 weeks (14 in each arm) receiving 100, 200, and 300 mg/kg/day, divided into two doses administered 12 hours apart for a total of 7 days, showed good tolerance with no effect on the mean arterial pressure or diastolic blood pressure [57].

### 6.2. Clinical Applications

#### 6.2.1. Bronchopulmonary Dysplasia with or Without Pulmonary Hypertension

L-CIT supplementation has been well studied in adults with pulmonary arterial hypertension. Kashani et al. demonstrated a significant clinical benefit in 25 adult patients with PH (idiopathic or Eisenmenger Syndrome) who received 1 g of L-CIT three times daily for two weeks [58]. Similarly, a 15.2% mean reduction was noted in the estimated pulmonary artery systolic pressure in sickle cell patients who were given L-ARG supplementation [59]. However, despite the burden of PH in premature infants with BPD and its associated adverse outcomes, few safe therapeutic options are available; while several studies strongly support that L-CIT is safe in stable premature infants, no efficacy trials have been conducted using L-CIT for the treatment of PH in this population.

The prevalence of BPD is increasing among extremely low birth weight infants (ELBW: birth weight < 1000 gms) in the US and Canada, where 40% of surviving infants born at <26 weeks’ gestation develop moderate-severe BPD [35,60,61,62]. Infants with BPD have a longer initial hospitalization, costing on average CAD 40,000 more than premature infants without BPD, and require more medical and specialized services throughout life, costing CAD 15,000 annually [63,64,65,66]. Current therapies, such as glucocorticoids, modulate inflammation and decrease the incidence of BPD in ELBW infants, but adversely affect growth, lung, and neurodevelopment [36,67,68]. Thus, safe and effective therapies to decrease the burden of BPD are needed.

Preterm infants have been found to have a lower mean plasma L-CIT concentration compared to older children [69,70]. Montgomery et al. evaluated 20 extremely low gestational age neonates with BPD in a cross-sectional study and found that infants with PH had lower median plasma L-CIT levels (21 μmol/L) compared to those who did not develop PH (36 μmol/L) [71]. This suggests the possible role of L-CIT as a biomarker for PH in premature infants with BPD. A case report published in 2018 describes a 5-month-old, ex-25-week infant weighing 700 g at birth, with ventilator-dependent severe BPD and associated PH despite iNO and oral sildenafil. He was started on a dose of 150 mg/kg/day of L-CIT, which was continued for 70 days. The infant was extubated from mechanical ventilation after 3 weeks of therapy and did not require intubation again until discharge. His brain natriuretic peptide levels decreased significantly over the first 4 weeks and remained stable until discharge. At the time of discharge, he required only 22–25% supplemental oxygen given intermittently during feeds [72]. Unfortunately, there are no published randomized controlled trials that have evaluated L-CIT as a potential therapeutic option for infants with PH secondary to BPD or BPD alone.

#### 6.2.2. Congenital Heart Disease

PH is a major complication after cardiac surgery in children with CHD and has been associated with adverse outcomes. Published studies have shown a mortality of 22.2–54.5% among children who developed PH post-cardiac surgery [73,74]. Infants undergoing cardiac surgery on cardiopulmonary bypass were found to have significantly decreased levels of L-ARG and L-CIT post-operatively. Thus, reduced bioavailability of NO precursors has been hypothesized to be a contributing factor to the increased risk of developing post-operative pulmonary arterial hypertension [75]. A randomized study enrolled 16 children undergoing reconstructive surgery for congenital heart disease to receive oral L-CIT supplementation or placebo [76]. L-CIT was administered orally in five doses of 3 g/m^2^ each, with a total dose of 15 g/m^2^. The first dose was administered during induction of anesthesia, the second upon arrival to the ICU, and subsequently every 12 h. The study revealed higher median plasma L-CIT levels in the perioperative L-CIT supplementation group compared to the control group. The levels were also higher at 12 h post-operatively in the supplementation group, a critical time point to prevent PH. Clinically, none of the children in the L-CIT supplemented group had mean pulmonary arterial pressures > 20 mmHg, as compared to 67% in the control group. No systemic hypotension was reported in the L-CIT group; in fact, the children showed greater stability of blood pressure during recovery and faster return to baseline blood pressure. A similar placebo-controlled randomized trial by Smith et al. with five perioperative doses (1.9 g/m^2^/dose) of oral L-CIT in children undergoing cardiac surgery found that children did not develop pulmonary arterial hypertension if they maintained a plasma L-CIT level > 37 μmol/L at 12 h post-operatively [77].

#### 6.2.3. Necrotizing Enterocolitis

In addition to prematurity, one of the important risk factors for BPD includes episodes of inflammation such as surgical NEC (sNEC). Infants with sNEC have a 3-fold increased incidence of BPD, excessive inflammation, and decreased absorptive capacity for many nutrients including amino acids leading to persistently low L-CIT levels [78,79,80]. Plasma L-CIT levels measured in 48 neonates with stage 2 or higher NEC identified lower levels in the first 24 h after the onset of NEC, suggesting a possible role of using L-CIT as an early biomarker for diagnosis, and a prognostic marker for recovery [81]. A study by Ioannou et al. reported significantly lower plasma L-CIT levels in neonates with NEC compared to healthy neonates of comparable gestational age and day of life. They also reported a sensitivity of 76% and specificity of 87% when using a cutoff of 17.75 μmol/L to identify neonates with NEC [82]. These studies highlight the possible role of plasma L-CIT levels in identifying at-risk neonates as well as in the early identification of the disease. The measurement of L-CIT levels on newborn screening dried blood spot on day 1 and 5 of life, and subsequently when the infant achieved full feeds, to identify neonates who subsequently developed NEC in a case-control study, did not show any difference between the two groups, limiting the use of the dried blood spot in identifying preterm neonates at risk for NEC [83]. Results from a Cochrane review, including three studies that enrolled 285 neonates, reported a moderate certainty of evidence of significant reduction in NEC and NEC-related mortality in preterm infants < 34 weeks treated with supplemental L-ARG [3]. However, there are no published trials on the effect of L-CIT supplementation in the prevention or as an adjunct in the treatment of NEC in preterm neonates.

#### 6.2.4. Mitochondrial Disease

Mitochondrial encephalomyopathy with lactic acidosis and stroke-like episodes (MELAS) syndrome is a congenital disorder that presents in the first decade of life. The exact mechanism of MELAS, though not fully understood, is hypothesized to involve NO deficiency (due to reduced precursor availability), mitochondrial energy failure, as well as microvascular angiopathy and impaired blood perfusion, all contributing to the pathogenesis of the disease [84]. A controlled trial by El-Hattab et al. found that children with MELAS had lower baseline levels of NO production, L-ARG and L-CIT flux, as well as plasma L-ARG concentrations compared to healthy counterparts. L-CIT supplementation in MELAS patients increased NO production, L-ARG and L-CIT flux, plasma L-ARG and L-CIT concentrations, and de novo synthesis rates of L-ARG, which was not observed in patients treated with L-ARG supplementation [85]. The improved benefit of L-CIT over L-ARG was believed to be due to the better absorption and systemic bioavailability of oral L-CIT over L-ARG. Observational studies have also shown that supplementation with NO precursors resulted in reduced stroke-like symptoms as well as decreased severity and frequency of such episodes, as well as the improvement in lactic acidosis [84,86]. These findings highlight the possible therapeutic role of L-CIT as an effective treatment for mitochondrial metabolic disorders associated with decreased NO bioavailability.

## 7. Implications for Future and Ongoing Research

First and foremost, it is essential that clinical trials are performed in the target population to determine tolerance, safety, and pharmacokinetics of any pharmacological intervention given to premature infants to mitigate the burden of disease, specifically BPD with or without PH. Diseases that are initiated by inflammation and oxidative stress adversely affecting lung development. A Phase-I study assessing safety and pharmacokinetics/pharmacodynamics (PK/PD) of oral L-CIT supplementation in preterm infants with BPD ± PH and NEC is currently ongoing at The Hospital for Sick Children, Toronto, ON, Canada, (ClinicalTrials.gov ID: NCT05636397). This PK/PD trial will provide important information on infants with evolving BPD by identifying safe doses for randomized controlled trials, which can be used in at-risk infants to help translate this novel therapy into clinical practice.

## 8. Conclusions

L-CIT supplementation appears to be a safe, well-tolerated, and promising new therapy for the treatment of a variety of neonatal conditions, particularly in BPD-associated PH and NEC. Future randomized controlled trials will be critical to evaluate the efficacy of L-CIT supplementation in maintaining L-ARG and, thus, NO bioavailability, to reduce progressive lung disease. This could have a significant impact on reducing the burden of these diseases, particularly in the extremely preterm population, who are at highest risk.
